# Consecutive intra-gingival injections of lipopolysaccharide and butyric acid to mice induce abnormal behavior and changes in cytokine concentrations

**DOI:** 10.1186/s12974-020-02008-8

**Published:** 2020-11-05

**Authors:** Takamitsu Tsukahara, Atsushi Toyoda, Takahiro Kawase, Shin-ichi Nakamura, Kuniyasu Ochiai

**Affiliations:** 1 Kyoto Institute of Nutrition and Pathology, Kyoto, Japan; 2 College of Agriculture, Ibaraki University, Ibaraki, Japan; 3 United Graduate School of Agricultural Science, Tokyo University of Agriculture and Technology, Tokyo, Japan; 4 Nihon University School of Dentistry, Tokyo, Japan

**Keywords:** Abnormal behavior, *n*-Butyric acid, Lipopolysaccharide, Mouse model, *Porphyromonas gingivalis*

## Abstract

**Background:**

Periodontopathic bacteria such as *Porphyromonas gingivalis* produce several metabolites, including lipopolysaccharide (LPS) and *n*-butyric acid (BA). Past work suggested that periodontal infection may cause cognitive impairment in mice.

**Aims:**

To elucidate the mechanisms by which metabolites such as LPS and BA, resulting from *Porphyromonas gingivalis* activity, induce immunological and physiological abnormalities in mice.

**Methods:**

In the present work, 28 male ICR mice were placed in an open-field arena and the total distance (cm/600 s) they covered was recorded. Based on their moving distances, mice were divided into 4 groups (*n* = 7) and injected the following substances into their gingival tissues for 32 consecutive days: saline (C), 5 mmol/L of BA (B), 1 μg/mouse of LPS (L), and BA-LPS (BL) solutions. Distances covered by mice were also measured on days 14 and 21, with their habituation scores considered as “(moving distance on day 14 or 21)/(moving distance on day 0)”. Afterwards, mice were dissected, and hippocampal gene expression and the concentrations of short-chain fatty acids, neurotransmitters and cytokines in their blood plasma and brains were analyzed. In addition, mouse brain and liver tissues were fixed and visually assessed for histopathological abnormalities.

**Results:**

Group BL had significantly higher habituation scores than C and B on day 14. LPS induced higher habituation scores on day 21. LPS induced significant decreases in the mRNA levels of interleukin (IL)-6 and brain-derived neurotrophic factors, and an increase in neurotrophic tyrosine kinase receptor type 2. In both plasma and brain, LPS induced a significant acetate increase. Moreover, LPS significantly increased acetylcholine in brain. In plasma alone, LPS and BA significantly decreased monocyte chemoattractant protein 1 (MCP-1). However, while LPS significantly decreased tyrosine, BA significantly increased it. Lastly, LPS significantly decreased IL-6 and tumor necrosis factor in plasma. No histopathological abnormalities were detected in liver or brain tissues of mice.

**Conclusion:**

We showed that injections of LPS and/or BA induced mice to move seemingly tireless and that both LPS and BA injections strongly induced a reduction of MCP-1 in blood plasma. We concluded that LPS and BA may have been crucial to induce and/or aggravate abnormal behavior in mice.

## Background

Gingivitis and periodontitis are periodontal diseases caused by microorganisms such as *Prevotella* spp. [1], *Fusobacterium* spp. [1, 2], and *Porphyromonas gingivalis* (Pg) [3], which can be found in the plaque biofilm surrounding the teeth [3]. These periodontopathic bacteria produce several secondary metabolites, including lipoproteins [4], lipopolysaccharide (LPS) [5] and *n*-butyric acid (BA) [1, 6, 7]. Common signs of chronic periodontal diseases include gingival erythema, edema, and periodontal pockets [5], which lead to destruction of supporting connective tissue and alveolar bone [3], ultimately resulting in tooth loss [4]. In a previous study, short-chain fatty acids (SCFA), such as BA, produced by pathogenic oral bacteria, had been associated with the inhibition of epithelial cell growth in vitro [2]. In addition, BA within the periodontal pockets, built by bacteria during periodontitis, compromised the expression of adhesion molecules by gingival epithelial cells [8], induced systemic inflammation [7] and indirectly promoted the progression of oral cancer [9].

Past work suggested that the release of pro-inflammatory cytokines, such as TNF-α and IL-6, due to periodontal infection, may have caused cognitive impairment in the brains of mice [5]. At these premises, we previously demonstrated that intra-gingival injections of BA-induced oxidative stress in blood mitochondria [6]. More recent evidence also seemed to link periodontitis caused by periodontopathic bacteria with the development of Alzheimer’s disease (AD) [10, 11]. However, up to date, a clear understanding of the biology and pathogenic mechanisms of the microbiome associated with the onset of AD remains elusive.

In the present study, we aimed to follow up our previous work and focus on the mechanisms of the metabolites produced by Pg such as LPS and BA, which induce immunological and physiological abnormalities. To achieve that, we injected LPS and/or BA to the gingival tissues of experimental mice, assessed the production of inflammatory cytokines and metabolites and evaluated the behavior of the mice.

## Methods

### Animals and experimental settings

Twenty-eight 14-week-old male ICR mice were purchased from Japan SLC (Shizuoka, Japan) and given a commercial AIN-93G pelleted feed (Oriental Yeast, Tokyo, Japan) throughout the study. All animals were housed in plastic cages (cage size 345 × 403 × 177 mm; CLEA Japan, Tokyo, Japan) with wood-shaving bedding (Pure Chip; Shimizu Laboratory Supplies, Kyoto, Japan) and maintained in an air-conditioned room (25 °C) in a 12-h-light/12-h dark cycle at the Kyoto Institute of Nutrition & Pathology (iNP, Kyoto, Japan). Food and water were provided to mice *ad libitum* throughout the experimental period. The handling of mice was carried out in accordance with the guidelines for animal studies of iNP (Approval number, 16026NU).

Lipopolysaccharide isolated from Pg and sodium *n*-butyrate were obtained from InvivoGen (San Diego, CA, USA) and FUJIFILM Wako Chemicals (Osaka, Japan), respectively. Both compounds were dissolved in and diluted with sterile saline, and the diluents were stored at – 20 °C until further use.

### Habituation test

In the present study, to assess the habituation abilities of the experimental mice, an open-field arena was used (500 × 500 × 400 mm; O’Hara & Co., Tokyo, Japan). The habituation test procedure was as that previously described [12]. The total distance (cm/600 s) traveled by mice was measured by TimeOFCR1 software (O’Hara & Co.) on days 0, 14, and 21, and the habituation score (%) was calculated as 100 × (moving distance on day 14 or 21)/(moving distance on day 0).

### Experimental design

**Fig. 1:**
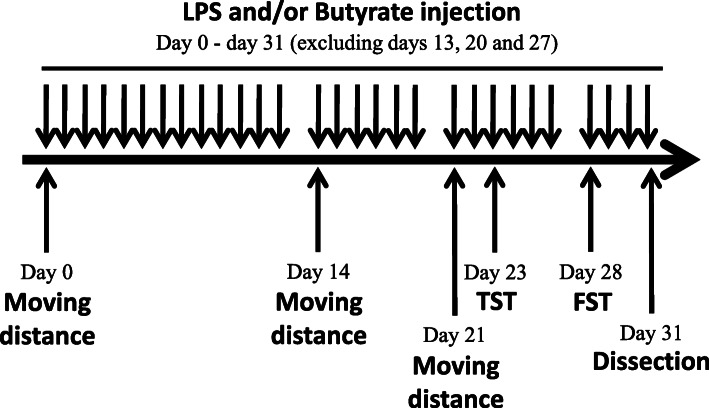
Experimental design of this study. TST: Tail suspension test, FST: Forced swim test

**Fig. 2:**
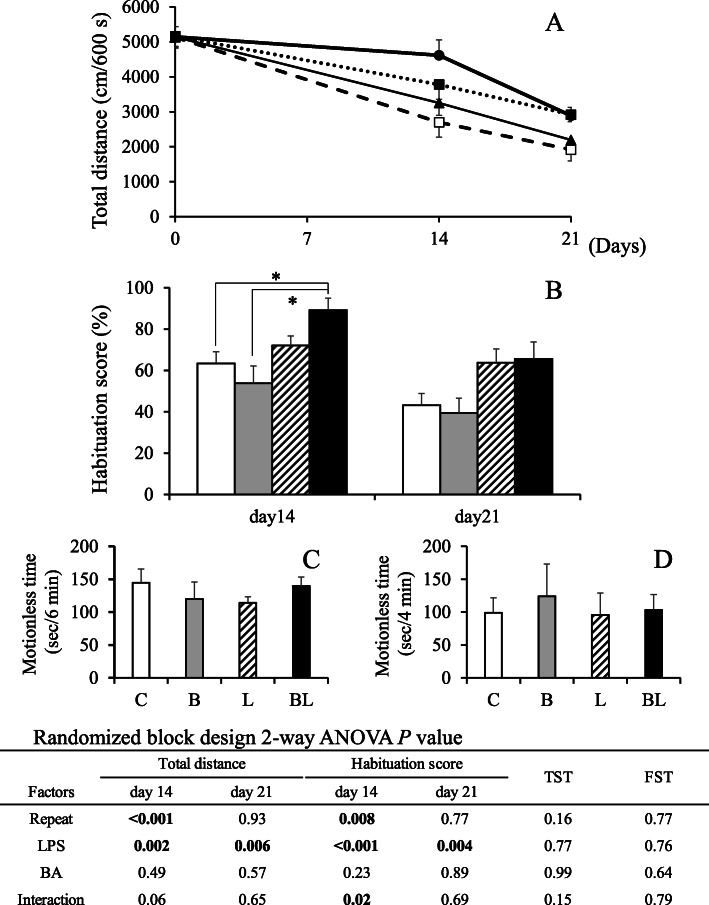
Effect of lipopolysaccharide (LPS) and/or n-butyric acid (BA) injection on mouse behavior. Closed triangles and open bars: Control (group C). Open squares and gray bars: BA injection (group B). Closed squares or slash bars: LPS injection (group L). Closed circles or closed bars: BA and LPS injection (group BL). **a** Total moving distance on days 0, 14, and 21. **b** Habituation score calculated by the total distance on days 0, 14, and 21. The asterisks represent significant differences (*P* < 0.05) between groups. **c** Immobility time in TST. Panel **d**: Immobility time in FST

The complete timetable of the experimental procedures is shown in Fig. 1. After 19-day acclimatization, mice were placed in the open-field arena individually, and their moving distances were recorded (day 0). Depending on the distance they covered (Fig. 2a), mice were sorted and equally divided (*n* = 7) into treatment-free saline (C), BA (B), LPS (L), and BA-LPS (BL) injection groups. Newly created experimental groups were then housed in separate plastic cages but maintained in the aforementioned room and conditions. Using a Hamilton Microliter Syringe 700 (Hamilton Company Japan, Tokyo, Japan), 5 mmol/L of BA and 1 μg/mouse of LPS were injected into the gingival tissues of groups B and BL and groups L and BL, respectively. Mice in group C were with injected saline only. For all mouse groups, the total volume of the treatment/control solution injected into the gingival tissues, equally divided for the right and left sides, was 10 μL/day. The solutions were injected at 11:00 for 32 consecutive days with some exceptions. Due to holidays, it was not possible to carry out the injection procedures on days 13, 20, and 27. The open-field test was also imposed on all mice on days 14 and 21. To test the behavior of mice, the tail suspension test (TST) and the forced swim test (FST) were imposed on mice on days 23 and 28, respectively. The procedures of behavioral tests were the same as those described elsewhere [13].

### Dissection

On day 31, dissection of mice started 4 h after the injection of the LPS and/or BA solutions. All mice were euthanized by exsanguination under deep anesthesia with an intraperitoneal injection of sodium medetomidine (300 μg/kg B.W.; Medetomin, Meiji Seika Pharma, Tokyo, Japan), midazolam (4.0 mg/kg B.W.; Dormicum, Astellas, Tokyo, Japan), and butorphanol tartrate (5.0 mg/kg B.W.; Vetorphale, MeijiSeika Pharma). Before exsanguination, blood was collected from the abdominal vein, and immediately cooled to 4 °C. The first mouse was dissected at 10:00 am. In total, seven replicates from each mouse group (C, B, L, and BL) were dissected.

The skull of each mouse was incised and the brain removed, with the left and right hemispheres being separated immediately. The left hemisphere was stored at – 80 °C for the neurotransmitter and SCFA analyses. The right portion of hippocampus was soaked into RNA-later solution (Sigma-Aldrich Japan, Tokyo, Japan) overnight at 4 °C. The hippocampal samples were then stored at – 80 °C for subsequent RNA extraction. The right portion of prefrontal area was fixed with a 10% (v/v) neutralized formalin solution. The liver was also removed and the right median lobe was fixed with a 10% (v/v) neutralized formalin solution. The remaining section was stored at – 80 °C and used in a different study. Blood was centrifugated (3000×*g*, 10 min, 4 °C), and the plasma was collected and stored at – 80 °C until further analysis.

### Measurement of gene expression in hippocampus

The methods used for total RNA extraction and cDNA synthesis were the same as those previously reported [14].

Real-time polymerase chain reaction (PCR) was conducted using a Rotor-Gene 6200 (Qiagen, Tokyo, Japan). Primers and TaqMan probes used in this study are listed in Table 1. Optimal primers and probes were designed using freely available online tools (https://www.roche-applied-science.com/) or selected from Bioresearch Technologies Japan (Tokyo, Japan). The methods employed for PCR analysis were the same as those previously described [12]. All genes analyzed for mRNA levels are also listed in Table 1.

**Table 1:** Primers and probes used in the present study

Gene name	Primers 5'-3'	Probe number	GenBank accession number
Interleukin-6 (*il6*)	F gatggatgctaccaaactggat	6	NM_031168.1
	R ccaggtagctatggtactccaga		
Interleukin-1 beta (*il1b*)	F gagaatgacctgttctttgaagttga	–	NM_008361.3
	R agatttgaagctggatgctctca		
	P accccaaaagatgaagggctgcttcc		
Tumor necrosis factor-alpha (*tnfa*)	F tgtctactgaacttcggggtga	–	NM_013693.2
	R gaagatgatctgagtgtgagggtct		
	P tccccaaagggatgagaagttccca		
Brain derived neurotrophic factor (*bdnf*)	F cacttttgagcacgtcatcg	42	*
	R tccttatggttttcttcgttgg		
Nerve growth factor transcript variant A (*ngf*)	F gtgcctcaagccagtgaaa	10	NM_013609.3
	R gaccacaggccaaaactcc		
Neurotrophin 3, transcript variant 1 (*nt3*)	F cgacgtccctggaaatagtc	29	NM_001164034.1
	R tggacatcaccttgttcacc		
Neurotrophic tyrosine kinase, receptor, type 1 (*trka*)	F tgtccaagtcagcgtctcc	20	NM_001033124.1
	R aaggggatgcaccaatgat		
Neurotrophic tyrosine kinase, receptor, type 2, transcript variant 1 (*trkb*)	F tgcccagagcaggataagat	76	NM_001025074.2
	R aaagtccttgcgtgcattgt		
Glyceraldehyde 3-phosphate dehydrogenase (*gapdh*)	F ggtgtcttcaccaccatgga	–	NM_008084.2
	R cagaaggggcggagatgat		
	P aaggccggggcccacttgaa		

Listed probe numbers indicate the product number of the Universal ProbeLibrary Set, Human and Extension Set sold by Roche Applied Science

*The primer sets of BDNF were designed in the same manner as previously described (Tsukahara et al. 2019)

Primers and probes of IL-1β, TNF-α and GAPDH were designed and synthesized by Bioreseach Technologies Japan (Tokyo, Japan)

### Measurement of the concentrations of short-chain fatty acids in blood plasma and brain tissues

The concentrations of SCFA in blood plasma and brain tissues were measured by gas chromatography coupled with mass spectrometry. One hundred milligrams of the mouse brain hemisphere sample was homogenized in 500 μL of 0.2 mol/L hydrochloric acid using a Micro Smash MS-100 apparatus (3000 rpm, 30 s; TOMY, Tokyo, Japan). The supernatant was collected after centrifugation (20,000×*g*, 15 min, 4 °C). SCFA were extracted from the supernatant and blood plasma collected prior to dissection. The SCFA extraction procedure and analysis methods were the same as those previously described [15, 16].

### Measurement of the concentrations of neurotransmitters in blood plasma and brain tissues

The concentrations of neurotransmitters such as acetylcholine (Ach), adrenaline, dopamine (DA), gamma amino butyric acid (GABA), kynurenic acid (KA), 3-hydroxykynurenine (3-HK), serotonin (5-HT), tryptophan (Trp), and tyrosine (Tyr) in blood plasma and brain tissues were measured using an ultra-pressure liquid chromatography apparatus equipped with a binary solvent manager, an autosampler, a column heater, and tandem mass spectrometry (Acquity TQD UPLC-MS/MS; Nihon Waters, Tokyo, Japan). The measurement procedure was the same as that previously described [17].

### Measurement of inflammatory cytokines in blood plasma

The concentrations of inflammatory cytokines such as interleukin (IL)-6, IL-10, IL-12 p70, interferon (IFN)-γ, monocyte chemoattractant protein (MCP)-1, and tumor necrosis factor (TNF) in blood plasma were measured by cytometric bead array (Mouse Inflammation CBA kit; Nihon Becton & Dickinson; Tokyo, Japan), as per the manufacturer’s instructions.

### Measurement of the concentration of corticosterone in blood plasma

To measure the levels of stress and memory impairment, the concentration of corticosterone in blood plasma was measured using a commercial ELISA kit (YK240 Corticosterone EIA Kit; Yanaihara Institute Inc., Shizuoka, Japan), as per the manufacturer’s instructions.

### Histopathological study

Fixed brain and liver samples were embedded into paraffin wax and cut into 4-μm-thick serial paraffin sections, which were then stained with hematoxylin and eosin. Histopathological abnormalities were assessed with a light microscope (BX51; Olympus, Tokyo, Japan). Evidence of amyloid deposition, extramedullary hematopoiesis, focus-of-altered hepatocytes, hepatocellular hypertrophy, or focal necrosis of hepatocytes was assessed in liver tissue samples. The number of glial cells and the level of infiltration of inflammatory cells were also assessed in brain tissue samples.

The aforementioned pathological abnormalities were scored by the following criteria: 0, normal; 1, slightly or locally abnormal; 2, moderately abnormal; 3, severely abnormal [18].

### Statistical analyses

A randomized block-design 2-way ANOVA (factors: BA and LPS) was used to analyze the differences between the means of the data from the habituation scores, the TST and FST tests, mRNA expression, and the concentrations of neurotransmitters, SCFA and corticosterone in plasma and tissues. Pathological scores were also analyzed with this method. When the interaction was significant, a randomized block-design one-way ANOVA was used to analyze differences between groups, Similarly, when an interaction effect in the habituation scores recorded on day 14 was detected by the two-way ANOVA, one-way ANOVA was used to analyze the data. In all statistical analyses, differences between the means were considered significant if *P* < 0.05. Outliers in the analyzed parameters were rejected using the Smirnov–Grubbs test. All data were analyzed using StatLight 2000 (Yukms, Kawasaki, Japan), an add-in application for Microsoft Excel© (Microsoft, Seattle, WA, USA). All values are shown as the means ± the standard errors of the means.

## Results

### Habituation and behavior tests

By days 14 and 21 (Fig. 2a, b), the data from the randomized block analysis showed that total distances and the habituation scores were significantly affected by the continuous injections of LPS into the gingival tissues. Mice injected with both BA and LPS (group BL) had significantly higher habituation scores than those in groups C and B. These results indicated that, unlike mice receiving injections of saline solution, injections of both BA and LPS induced mice to move seemingly tireless, even though the area of movement (the open-field arena) was the same as that used for control mice.

In contrast, although all mice underwent the TST (Fig. 2c) and FST (Fig. 2d) tests, no differences in the immobility times were found between the experimental mouse groups, after the test scores were calculated.

### Effect of treatments on the mRNA level in hippocampus

**Fig. 3:**
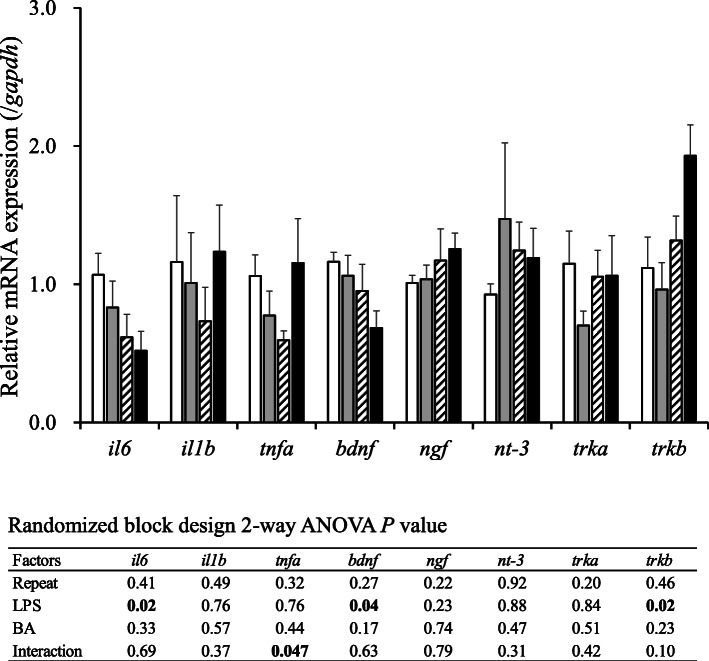
Effect of lipopolysaccharide (LPS) and/or butyrate injection on hippocampal mRNA expression. For further details, see Fig. 2

The mRNA expression levels of inflammatory cytokines, neurotrophic factors and their receptors in the hippocampus were compared between control and treatment mice (Fig. 3). LPS induced significant (*P* < 0.05) decreases in the mRNA levels of IL-6 and brain-derived neurotrophic factors (BDNF), but increased the mRNA level of neurotrophic tyrosine kinase receptor type 2 (TrkB) (Fig. 3). No other mRNA expression was affected by treatments.

### Concentration of short-chain fatty acids in blood plasma and brain tissues

Injections of LPS to mice significantly increased the acetate level in blood plasma, but BA remained unchanged (Table 2). Similarly, LPS injections to mice significantly increased the acetate level in brain tissues. Nonetheless, the concentrations of others SCFA in brain tissues of mice were too low to be detected by the analysis.

**Table 2:** Effects on the LPS and/or BA injection on the SCFA concentrations in plasma and brain tissues

Parameters	Groups	Plasma			Brain
		Acetate	Propionate	*n*-Butyrate	Acetate
SCFA concentrations (μmol/L)	C	199.8	12.1	1.61	1310.6
	B	172.8	16.1	1.07	1203.7
	L	595.6	24.6	1.18	1411.7
	BL	669.3	28.8	2.30	1487.1
	pooled SE	83.1	4.1	0.28	31.1
Randomized block design 2-way ANOVA *p* value	Repeat	0.29	0.93	0.87	0.93
	LPS	**0.01**	0.15	0.53	**0.003**
	BA	0.85	0.63	0.53	0.93
	Interaction	0.74	0.99	0.16	0.10

No concentration of propionate or *n*-butyrate was detected in the mouse brain samples

### Effect of treatments on the concentrations of neurotransmitters in blood plasma and brain tissues

LPS injections significantly *(P* < 0.05) increased the concentration of Ach in mouse brain tissues (Table 3). As for the concentrations of neurotransmitters in blood plasma, while LPS injections significantly *(P* < 0.05) decreased the concentration of Tyr, BA injections significantly *(P* < 0.05) increased it. No other neurotransmitters were significantly changed by the treatments. It is worth noting that the factor “repeat” showed that, except for Trp, GABA, and KA, the concentrations of the other neurotransmitters analyzed in brain tissues were significantly affected.

**Table 3:** Effect of the LPS and/or BA injection on the neurotransmitter concentrations in plasma and brain

Parameters	Groups	Brain							Plasma		
		Tyr	Trp	GABA	Ach	DA	5-HT	KA	Tyr	Trp	GABA
Neurotransmitter concentrations (μmol/L)	C	530	267	8,320	12.9	91.0	0.15	0.31	134	11.4	0.36
	B	605	234	8,070	8.7	87.6	0.15	0.35	210	10.2	0.36
	L	485	224	7,980	21.4	101.9	0.16	0.32	113	12.9	0.35
	BL	514	235	7,991	20.7	100.2	0.16	0.32	137	10.3	0.42
	pooled SE	22	9	103	2.1	5.2	0.01	0.01	12	1.1	0.05
Randomized block design 2-way ANOVA *p* value	Repeat	**0.04**	0.78	0.71	**0.003**	**<0.001**	**0.001**	0.31	**<0.001**	0.99	**<0.001**
	LPS	0.10	0.23	0.26	**0.01**	0.24	0.45	0.71	**0.02**	0.75	0.67
	BA	0.66	0.75	0.21	0.67	0.93	0.85	0.41	**0.007**	0.41	0.39
	Interaction	0.23	0.68	0.39	0.82	0.7	0.56	0.46	0.07	0.76	0.45

No concentration of 3-Hydroxy kynurenine or adrenaline was detected in the samples

The concentrations of Ach, DA, 5-HT and KA in blood plasma were too low to be detected by the analysis

*CO* control group, *BA* Butyrate group, *LP* Lipopolysaccharide group, *BL* Butyrate and lipopolysaccharide group, *Tyr* tyrosine, *Trp* tryptophan Trp, *GABA* gamma amino butyric acid, *ACh* acetylcholine, *DA* dopamine, *5-HT* serotonin, *KA* kynurenic acid

### Effect of treatments on the concentrations of inflammatory cytokines and corticosterone in blood plasma

**Fig. 4:**
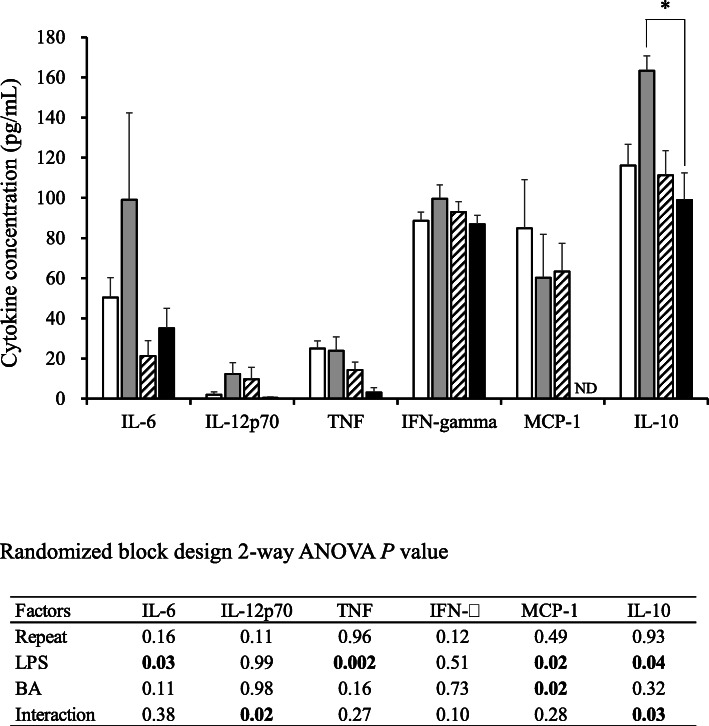
Effect of lipopolysaccharide (LPS) and/or butyrate injection on the concentrations of cytokines in blood plasma. For further details, see Fig. 2

The concentration of corticosterone in blood plasma did not change significantly between mouse groups (345.9 in group C; 274.7 in group B; 294.4 in group L; and 257.4 ng/mL in group BL). However, the concentrations of analyzed cytokines were changed by the treatments administered to mice. For example, the injections of BA decreased the concentration of MCP-1. Similarly, LPS injections significantly decreased the concentration of IL-6, TNF, and MCP-1 (Fig. 4). Finally, the sole injection of BA significantly (*P* < 0.05) increased the concentrations of IL-10, when compared with mice being injected with both LPS and BA (Fig. 4). The concentrations of IL-12p70 and IFN-γ did not significantly change between experimental groups.

### Histopathological study of the brain and liver tissues

Histopathological observations did not detect abnormal glial cells in brain tissues between experimental groups. Moreover, no abnormal infiltration of inflammatory cells was observed in brain tissues of mice. Likewise, liver abnormalities such as amyloid deposition, extramedullary hematopoiesis, focus of altered hepatocytes, hepatocellular hypertrophy, and focal necrosis of hepatocytes were not detected even marginally in the tissues of mice. These observations inferred that no significant tissue damage occurred in treated mice, when compared with control mice (data not shown).

## Discussion

The metabolites produced by periodontopathic bacteria that cause diseases, such as gingivitis and periodontitis, have been increasingly associated with systemic inflammation and memory impairment [19]. In the present work, we wanted to know whether injections of LPS and BA, metabolites usually produced by periodontopathic bacteria, could cause deleterious effects on the cognitive skills and behavior of mice. The present study was designed in such manner that 1 μg/mouse of LPS and/or 5 mmol/L BA were daily injected into the gingival tissues of mice to evaluate whether abnormal behavior occurred as a result. The concentrations of these injections were relatively lower when compared with those reported in previous studies where abnormal behavior was induced [19, 20]. In our previous study, we showed that 5 mmol/L of - BA and 1 μg/mL of LPS induced apoptosis of human peripheral blood mononuclear cells in vitro [21]. In the present work, we wanted to simulate physiological concentrations of LPS [22] and BA [23] similar to those produced by periodontopathic bacteria present in the gingival sulcus of a host experiencing abnormal behavior. We theorized that if relatively low concentrations of LPS and/or BA could induce abnormal behavior in mice, localized periodontitis could also induce similar behavior in humans.

Wu et al. report that “chronic systemic exposure to LPS (from Pg) initiates AD-like phenotypes, including learning and memory deficits” in middle-aged mice but not in young WT mice [19]. In the present study, mice receiving an injection of LPS or LPS with BA showed more activity than other mice, with longer strolls, especially during the first 14 days of the experimental period. For example, mice in groups L and BL were seemingly more tireless and group BL had a higher habituation score on day 14 than mice in other groups (Fig. 2b). Stimulation of the Ach concentration in the brain by LPS injections seemed to substantiate these observations (Table 3). In humans, similar traits are often associated with dementia and AD, which are characterized by systemic inflammation and neuronal loss [24]. Interestingly, on day 21, this deleterious effect was not observed in mice. Although a direct comparison cannot be made, in the present work, the abnormal behavior observed in mice was similar to that usually reported in humans suffering from dementia and AD, which, in the case of the present work, was likely caused by a synergistic and deleterious effect of BA and LPS on mouse brains. For example, the injections of LPS decreased not only the mRNA levels of neurotrophic factor BDNF, but also the concentration of pro-inflammatory cytokine IL-6 in the hippocampus (Fig. 3). Brain derived neurotrophic factor, a factor involved in the survival of neurons [25], is very active in areas associated with learning, memory and higher thinking, such as the cortex, basal forebrain and hippocampus [26]. Thus, it would be natural that a decrease in the expression of *bdnf* in mouse hippocampus would result in cognitive impairment in mice, as demonstrated in the present work. Furthermore, *trkb*, a receptor with high affinity against BDNF, was stimulated by LPS injections. This phenomenon was reasonably expected because hippocampal *bdnf* is associated with its *trkb* expression [27]. It remains equivocal, however, the reason inflammatory cytokine *il6* decreased after the LPS injections. Similarly, except for IL-12p70 and IFN-gamma, the concentrations of all cytokines in blood plasma of mice receiving LPS injections were found to be lower than in mice not having LPS injected (Fig. 4). Production of LPS caused by periodontal health problems has been shown to induce an increase in pro-inflammatory cytokines that in turn cause neuroinflammation and cognitive impairment, which ultimately may result in diseases such as dementia and AD [5, 24]. A possible explanation for this apparent ambiguity may be that LPS induces production of both pro- and anti-inflammatory cytokines, as it has been previously demonstrated in vitro [28], in order to keep an immune balance. In addition, certain cells may be more susceptible to the deleterious effect of LPS than others [29]. For example, Jones et al. [29] showed that unlike mouse alveolar macrophages, mouse gingival fibroblast cells were less responsive to stimulation by LPS from Pg, hence producing less pro-inflammatory cytokines such as IL-6 and MCP-1. Although our results are in agreement with the data from these previous studies, the scope of the present work was an inevitable limiting factor. Indeed, a broader approach could have further explained the systematic decrease of inflammatory cytokine production induced by the LPS administration, which is the reason we believe further investigation of the systematic inflammatory cytokine production is necessary. It is worth noting that behavior after injections of LPS has been previously shown to differ between mouse strains such as C57BL/6 and ICR (CD-1) [30]. Furthermore, Painsipp et al. [30] suggested that depression-like behavior depended on LPS susceptibility, that is to say, while LPS administration induces severe depression-like behavior in C57BL/6 mice, it does not in ICR mice. Thus, in the present work, we hypothesized that, by using an LPS-susceptible strain such as C57BL/6, we may have also inadvertently induced a more severe behavioral response than if we had used a different mouse strain. We suggest that the response of different mouse strains to LPS administration be investigated in the future.

It has been proposed that BA produced during periodontitis can cause systemic inflammation [7], via the production of pro-inflammatory cytokines such as MCP-1 [31]. However, in the present work, we found that while a combined injection of both LPS and BA inhibited the production of IL-10 (Fig. 4), a well-known anti-inflammatory cytokine [32], the sole injection of BA to mice induced a decrease in the concentration of pro-inflammatory MCP-1. IL-10 has been reported to play a role in downregulating the production of pro-inflammatory cytokines [33, 34]. Thus, based on the present results, it can be inferred that simultaneous injections of BA and LPS to mice induced a synergistic and adverse effect, as discussed above, causing a more severe inflammation than injections of only LPS or BA would have caused.

In the present work, in both blood plasma and brain tissues of mice, an increase in the concentration of acetate was detected, even though, under normal circumstances, the concentration of acetate in circulation is low (e.g., 199.9 μmol/L in group C) [35]. Elsewhere, acetate has been found to be produced during endogenous bacterial fermentation in the gut as a protective response [36]. In addition, acetate has also been shown to ameliorate the pro-inflammatory effect of LPS in vitro [37, 38]. Furthermore, the anti-inflammatory properties of SCFA, acetate included, have been compared with those of inflammation markers [39]. Therefore, a high acetate concentration induced by the LPS injection may have been a biological response in mice against inflammation. Recently, Kimura-Todani et al. suggested that a high release of acetate into the large intestine decreased the anxiety-like behavior of BALB/c mice [40]. Therefore, the habituation results in the present study could also be associated with a high production of acetate caused by LPS injections. This speculation remained unaddressed as we did not carry out the marble burying and elevated plus-maze tests that Kimura-Todani et al. did to determine anxiety-like behavior [40]. Therefore, it remains unclear whether the high concentrations of acetate found in samples were truly associated or not with the scores obtained from the habituation test. By contrast, in the present study, BA concentration did not increase either in blood plasma or brain, even after BA was injected to mice (Table 2). This result may suggest that although BA did not affect the mouse behavior directly, a yet unknown factor or factors induced by or associated with BA may have done so.

Clearly, using a habituation test, memory impairment was observed in mice injected with solutions of LPS and BA. However, in past work, to evaluate the behavior of mouse models of AD, different methods such as fear conditioning, radial arm maze and Morris water maze tests were used [41]. Therefore, in the present study, the use of the habituation test to evaluate the behavior of mice may have been a limiting and/or confounding factor. We plan to use other behavioral methods to test the behavior of mice receiving LPS and BA injections in future studies.

In the present study, the concentration of Tyr in blood plasma was affected by LPS and BA injections (Table 3). Since Tyr is known as a precursor to a number of neurotransmitters [42], the concentration of Tyr in circulation may be associated with abnormal behavior. However, except for Ach, no neurotransmitters were affected by either the LPS or the BA injections, hence the mediation of Tyr seemed to have been limited. In addition, it is noteworthy that in the present study, the concentrations of many neurotransmitters were shown as significant by the “repeat” factor (Table 3). While these data seem to suggest that the timing of euthanizing is important to estimate the true concentrations of neurotransmitters in brain tissues, the questions remain unanswered. We suggest that further investigation be also conducted to address these questions.

## Conclusions

Our data showed that injections of LPS and/or BA to mice caused abnormal activity such as seemingly tireless movement in an open-field arena. Several studies have already reported that LPS induces chronic and systemic abnormalities. Nonetheless, we theorized that BA and/or other metabolites may have also been just as crucial to induce and/or aggravate abnormal behavior in mice.

## Data Availability

Data and data analyses of this study are largely included in this article. Complete datasets used and/or analyzed in the current study are available from the corresponding author upon reasonable request.

## Abbreviations

Ach: Acetylcholine
AD: Alzheimer’s disease
BA: *n*-Butyric acid
BDNF: Brain-derived neurotrophic factor
DA: Dopamine
FST: Forced swim test
GABA: Gamma amino butyric acid
3-HK: 3-Hydroxykynurenine
5-HT: Serotonin
IL: Interleukin
KA: Kynurenic acid
LPS: Lipopolysaccharide
MCP: Monocyte chemoattractant protein
PCR: Polymerase chain reaction
Pg: *Porphyromonas gingivalis*
SCFA: Short-chain fatty acids
TrkB: Neurotrophic tyrosine kinase receptor type 2
Trp: Tryptophan
TST: Tail suspension test
TNF: Tumor necrosis factor
Tyr: Tyrosine
